# A randomized vagus nerve stimulation study demonstrates that serum aldosterone levels decrease with age in women, but not in men

**DOI:** 10.1038/s41598-023-40113-9

**Published:** 2023-08-30

**Authors:** Elisabeth Veiz, Susann-Kristin Kieslich, Dirk Czesnik, Christoph Herrmann-Lingen, Thomas Meyer, Julia Staab

**Affiliations:** 1 Department of Psychosomatic Medicine and Psychotherapy, University Medical Center, Göttingen, Germany; 2 Department of Neurology, University Medical Center, Göttingen, Germany; 3 German Center for Cardiovascular Research (DZHK), Partner Site Göttingen, Göttingen, Germany

**Keywords:** Biomarkers, Medical research

## Abstract

In this randomized, sham-controlled study, we explored the effects of acute transcutaneous vagus nerve stimulation (tVNS) on serum aldosterone in 20 younger (21–26 years) and 19 older (40–70 years) healthy participants. Blood samples were collected on two different days before and after a 20-min application of active tVNS at the inner tragus or sham stimulation of the earlobe. Irrespective of the stimulation mode, aldosterone levels decreased from pre- to post-stimulation in both the young (active: β = − 1.610 (− 2.855, − 0.365), p = 0.022; sham: β = − 0.857 (− 2.102, 0.388), p = 0.257) and the old cohort (active: β = − 1.969 (− 3.234, − 0.703), p = 0.005; sham: β = − 1.334 (− 2.600, − 0.069), p = 0.063). Although this decline was significant during active tVNS, the difference in estimated β-coefficients between active and sham stimulation was not statistically significant in either cohort. Nevertheless, aldosterone concentrations showed a significant interaction effect between sex and age (p = 0.001). Among all study participants, younger women (23.3 ± 1.6 years) had the highest mineralocorticoid levels (pre active: 172.1 ± 102.0 pg/ml, pre sham: 214.3 ± 82.3 pg/ml), whereas the lowest were observed in older females (59.4 ± 9.4 years) (pre active: 104.9 ± 85.8 pg/ml, pre sham: 81.1 ± 53.8 pg/ml). This post hoc analysis did not suggest that active auricular tVNS reduces serum aldosterone levels compared to sham stimulation in healthy subjects. However, serum aldosterone levels differed among subjects depending on their age and sex, irrespective of tVNS.

## Introduction

Worldwide, psychosocial stress is an important risk factor for the development of cardiovascular diseases^1,2^. Underlying pathophysiological mechanisms include changes in the neuroendocrine system mainly within the hypothalamo-pituitary-adrenocortical (HPA) axis as well as a dysregulated autonomic nervous system^2–4^. It has been well established that exposure to psychosocial stressors results in the secretion of corticotropin-releasing hormone (CRH) from the paraventricular nucleus and the subsequent release of adrenocorticotrophic hormone (ACTH) from the pituitary gland. This leads to the production of glucocorticoids and their release from the adrenal cortex into the circulation^3,5,6^. However, much less is known about the link between psychosocial stress and the mineralocorticoid hormone aldosterone, which shows structural similarity to cortisol. These steroid hormones are synthesized in the adrenal cortex and bind as intracellular ligands to homologous nuclear receptors. As an essential component of the renin–angiotensin–aldosterone system (RAAS), aldosterone plays an important role in maintaining electrolyte and water homeostasis in the distal tubules and collecting ducts of the nephron by stimulating the reabsorption of sodium and excretion of potassium^4,7^. Excessive aldosterone levels thus promote hypertension and cardiovascular injuries such as myocardial hypertrophy, fibrosis, and atherosclerosis^8^.

Elevated aldosterone concentrations have been linked to an impaired baroreflex sensitivity, which is another risk factor for cardiovascular diseases^9–11^. The baroreceptor sensitivity is modulated by the parasympathetic nervous system, which innervates the heart and other visceral organs via the vagus nerve. Currently, transcutaneous vagus nerve stimulation (tVNS) is increasingly being investigated for the treatment of depression and other stress-related psychiatric disorders such as post-traumatic stress disorder^12^. Depressed patients were shown to have altered aldosterone concentrations in the circulation. Two studies revealed significantly higher aldosterone levels in depressed subjects compared to non-depressed controls^13,14^. Similarly, a cross-sectional study in 1743 participants reported a significant association between higher aldosterone levels and the combination of depressive symptoms and living alone^15^. However, suicidal patients showed significantly lower aldosterone concentrations compared to non-suicidal patients and healthy controls, which may have been a consequence of sustained RAAS hyperactivity during the acute depressive phase^16^. In a study with 521 middle-aged women, participants suffering from chronic post-traumatic stress disorder had lower aldosterone levels compared to subjects without trauma^17^. A cross-sectional study with 3092 participants from the general population found significantly increased renin and unaltered aldosterone levels in traumatized subjects with and without post-traumatic stress disorder^18^. Recent findings suggest that acute stress induced by the Trier social stress test resulted in increased plasma aldosterone and renin concentrations in young healthy men^19,20^. Likewise, hypertensive men showed a significantly higher increase in aldosterone and renin levels in response to acute stress compared to normotensive men^21^.

Given that aldosterone concentrations are increased during stress and depression, tVNS may influence aldosterone secretion due to its reported anti-depressant and stress-relieving effects. Therefore, we performed a post hoc analysis using data from a sham-controlled, cross-over study to explore the effects of acute tVNS on the aldosterone concentrations in both a younger and an older cohort of healthy participants. We hypothesized that the aldosterone concentration decreases upon tVNS intervention in both genders, but not during sham treatment.

## Materials and methods

### Study participants

The study cohort consisted of 20 younger (range 21–26 years, 10 women) and 19 older healthy participants (range 40–70 years, 9 women). Before study inclusion, a standard interview about participant’s medical history was conducted to ensure that probands were free of any somatic or mental diseases and did not take any medications, except contraceptives. Further, subjects were not allowed to consume caffeine, nicotine and alcohol at least three hours before each scheduled recording. All experiments were performed in accordance with the Declaration of Helsinki and were approved by the local ethics committee at the University Medical Center Göttingen (protocol number: UMG 27/7/18). Informed consent was obtained from all participants included in the study.

### Experimental protocol

**Figure 1:**
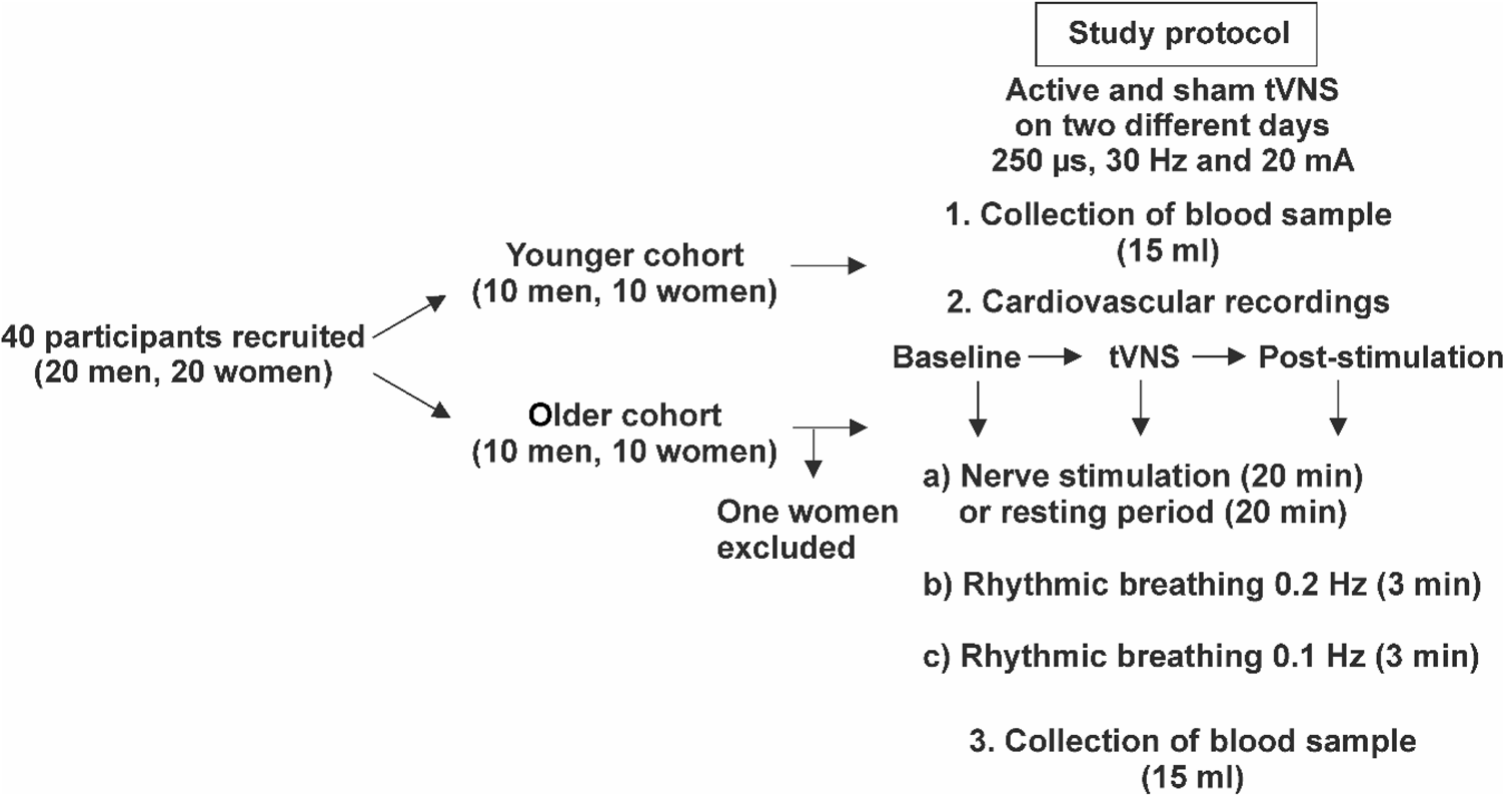
Experimental protocol of the randomized crossover study. Study participants received the application of sham and active tVNS on two different days. Following pre-experimental blood collection, younger participants were subjected to electrical nerve stimulation on the forearm, whereas older participants rested for 20 min. Afterwards, two rhythmic breathing tasks at 0.2 Hz and 0.1 Hz were performed for three minutes each. The consecutive sequence from 1 to 3 was repeated three times. The experimental day ended with the second blood collection.

Each participant underwent one active and one sham tVNS stimulation in a randomized order on two different days, with a minimum interval of 24 h in between. On each day, electrocardiographic and blood pressure recordings were performed continuously in a relaxed and seated position for the duration of the experiment using the Task Force Monitor (CNSystems, Graz, Austria). The study protocol was separated into three different tasks (forearm stimulation/resting period, rhythmic breathing at 0.2 Hz, and rhythmic breathing at 0.1 Hz). These tasks were repeated three times, before (baseline), during and immediately after the active or sham tVNS (post-stimulation) (Fig. 1). Electrical stimulation of the median nerve at the forearm was initially planned for all participants to investigate tVNS effects on peripheral nerve excitability^22^. During forearm stimulation younger probands reported recurrent discomfort or mild pain. Therefore, this task had to be discontinued for older subjects, and the experimental protocol was changed to a resting period of similar length. The rhythmic breathing tasks were included to control for respiratory sinus arrhythmia. On each day, 15 ml of venous blood were collected before and after the experimental period. Collected blood samples were cooled to 4 °C, centrifuged and stored at -80 °C on the same day until further processing. A more detailed description of the experimental protocol can be found elsewhere^22^.

### Transcutaneous vagus nerve stimulation

All subjects received active and sham tVNS treatment in a crossover design. During their first visit, participants were randomized by throwing a dice with respect to the order of active or sham intervention, resulting in a balanced assignment. Subjects received all necessary information for their participation in the study, except that the nature of the vagus nerve stimulation and the exact electrode placement were not mentioned to ensure their blindness. The inner tragus of the left ear was used as the active stimulation site, while the left earlobe served as the sham control. Previous skin preparations included a cleaning with disinfectant alcohol and the application of conductive gel. The Easy Tens + device with customized bipolar clip electrodes (both obtained from body clock, London, UK) was used to deliver continuous biphasic, rectangular pulses with a pulse width of 250 µs and a frequency of 30 Hz. The current amplitude was fixed on 20 mA, which was perceived by the subjects, but never reported as being painful.

### Aldosterone measurement

Collected blood samples were centrifuged at 2000 g and 10 °C for 10 min and serum probes were stored at − 80 °C until evaluation. Serum aldosterone concentrations were determined twice according to the manufacture’s protocol using the ELISA kit (RE52301) from Tecan (Männedorf, Switzerland). The optical density of the samples and standard solutions used as references were measured at 450 nm. Quantified absorption levels were averaged and standard curves were calculated using a four-parameter logistic curve fit with the freely available AssayFit Pro add-in (Version 1.41, Nijmegen, Netherlands) for Microsoft Excel.

### Statistical analysis

Changes in serum aldosterone concentrations between the pre- and post-stimulation phase were tested with linear mixed models (LMMs) using a customized design matrix. Statistical models were implemented in Python using the module statsmodels^23^. Inter-subject variability was taken into account by adding a group variable as a random effect. A fixed main effect of sex was added to address potential differences between men and women. Furthermore, body-mass index (BMI) was entered as a covariate, since previous studies had suggested a link between BMI and aldosterone levels^24–26^. Since the assessment of LMM residuals revealed a violation of normality, aldosterone concentrations were transformed using the Yeo-Johnson power transformation implemented with the Python module SciPy^27,28^. Estimated model coefficients are reported as β. To verify that changes in aldosterone level were different between the active and sham conditions, a post hoc F-test was applied to the estimated LMM coefficients. Data transformation and statistical analysis regarding potential tVNS effects were performed separately for the younger and older groups, since the difference in the experimental protocols made between-group comparisons difficult to interpret. Age and sex effects on aldosterone levels independently of tVNS were analyzed with an LMM using the entire transformed data set of both the younger and older cohorts with four repeated measurements per subject. Baseline sex differences in the mean arterial blood pressure (mBP) were separately tested for the young and old cohorts using LMMs, while group-specific sex differences in age and BMI were tested using the Mann-Whitney U test. Since the statistical analysis was part of a post hoc evaluation, all p-values were adjusted for multiple testing using the Benjamin-Hochberg method with an alpha level of 5%. Subsequently, only the corrected p-values were reported. Excel (Microsoft Office Professional Plus 2019) was used for the calculation of serum aldosterone concentrations, and statistical analysis was conducted with Python (version 3.8.10). All figures were created using the Python modules Seaborn 0.11.1^29^ and Matplotlib 3.4.2^30^ and edited using CoralDRAW 2021 (Corel Corporation, Ottawa, ON, Canada).

## Results

### Baseline characteristics

Females and males in the young cohort had a similar age distribution with a median age of 23 years, while the median in the old cohort was 7.5 years higher in the females compared to their male counterparts. In both age groups, male participants had a non-significantly higher BMI compared to female subjects. On both experimental days, the mBP of older men was markedly higher compared to older women (p < 0.001, Table 1). In contrast, the difference in mBP between young men and young women was less distinct, since younger men had significantly higher mBP on the day of sham stimulation (p = 0.002) and a similar trend on the day of active tVNS (p = 0.061, Table 1). Table 1 describes the baseline characteristics between men and women in both cohorts.

**Table 1:** Baseline characteristics of male and female participants for the two experimental procedures (tVNS and sham treatment).

	Stimulation			Sex effect (Men)	p-value
Young cohort		Men (n = 10)	Women (n = 10)	Coeff (95%CI)	
Age (years)		23 (22–26)	23 (21–26)		0.454
BMI (kg/m^2^)		22.8 (19.2–26.0)	20.9 (18.6–23.2)		0.124
mBP (mmHg)	Active	82.8 ± 8.1	74.8 ± 9.1	7.99 (0.51,15.47)	0.061
mBP (mmHg)	Sham	84.3 ± 7.9	76.2 ± 11.2	8.12 (3.45,12.79)	0.002

Old cohort		Men (n = 10)	Women (n = 9)		
Age (years)		56.5 (40–70)	64 (43–70)		0.377
BMI (kg/m^2^)		28.1 (24.6–49.1)	23.6 (18.7–29.0)		0.414
mBP (mmHg)	Active	101.7 ± 11.3	80.5 ± 12.2	21.24 (17.46, 25.01)	< 0.001
mBP (mmHg)	Sham	98.4 ± 15.5	83.4 ± 12.4	14.97 (12.68, 17.27)	< 0.001

Shown are the median age and the median body-mass index (BMI) with the respective value range. Mean arterial blood pressure (mBP) is displayed as the mean ± standard deviations during the first baseline measurement. Baseline sex differences in mBP were tested using a linear mixed model, while sex differences in age and BMI were tested using Mann-Whitney U test. Estimated coefficients, representing the effect of being male, are depicted with the 95% confidence interval (95%CI).

### Aldosterone concentrations in relation to tVNS treatment

**Figure 2:**
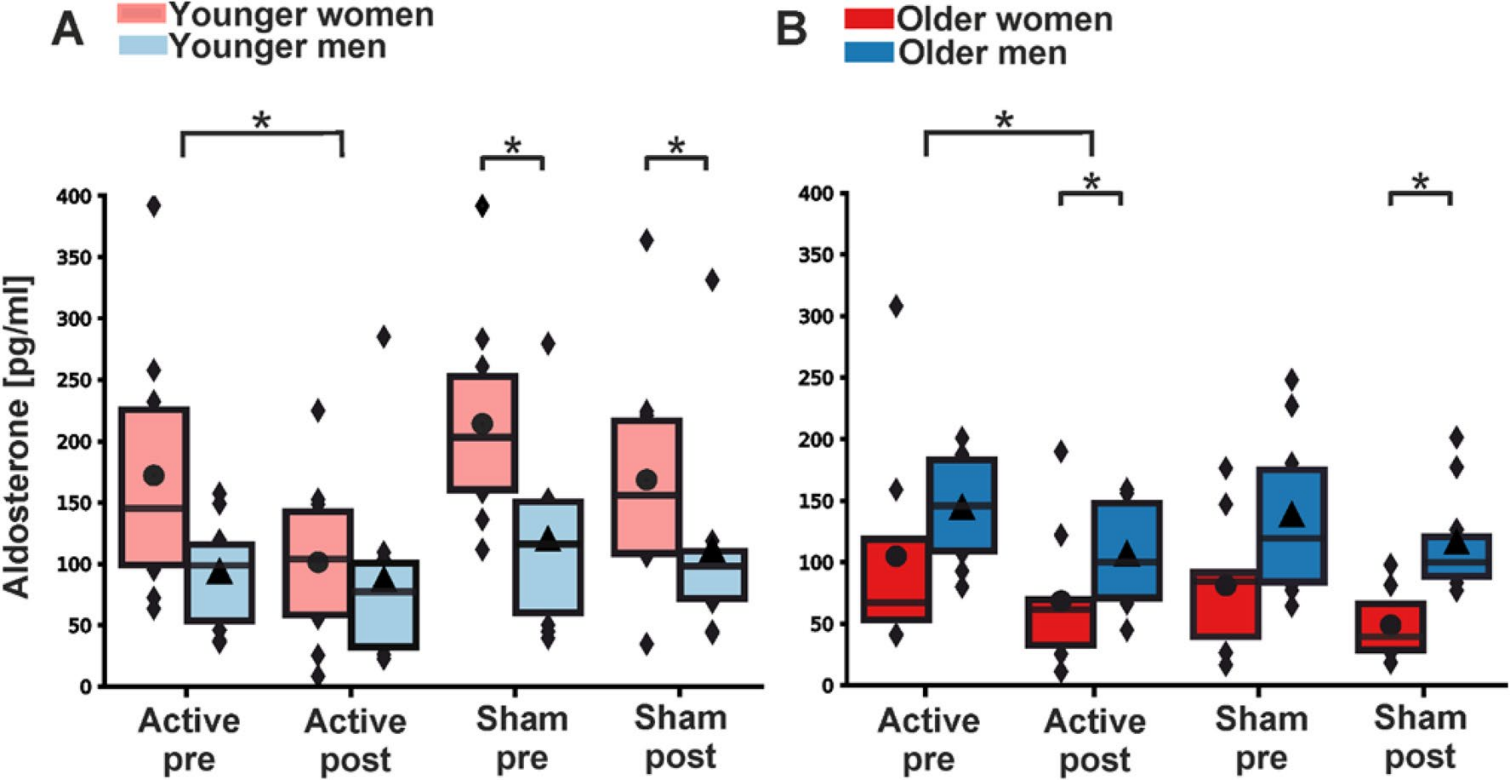
Boxplots of untransformed aldosterone concentrations before (pre) and after (post) intervention. (**A**) Aldosterone levels during active tVNS and sham treatment for men and women from the younger cohort. (**B**) Aldosterone levels during active tVNS and sham treatment for men and women from the older cohort. The colored boxes represent 50% of all the data points (red: female, blue: male). Black dots depict average values for females, while triangles depict the average value for males. The black lines depict the median of the respective distribution plots. Significant differences are shown between the pre- and post-stimulation conditions (linear mixed models) and the comparison between males and females within the pre- and post-stimulation condition (Mann-Whitney U test). *p < 0.05.

In the young cohort, aldosterone concentrations decreased significantly from pre- to post-stimulation after active tVNS (β = − 1.610 (− 2.855, − 0.365), p = 0.022) and not significantly after sham treatment (β = − 0.857 (− 2.102, 0.388), p = 0.257) (Table 2 and Fig. 2A). As aldosterone levels decreased during both active tVNS and sham stimulation, the F-test between the estimated β-coefficients revealed no significant difference (F_1,76_ = 0.702, p = 0.452). Similar results were found in the old cohort, in which aldosterone concentrations dropped significantly after active tVNS (β = − 1.969 (− 3.234, − 0.703), p = 0.005) compared to a trend towards decline following sham treatment (β = − 1.334 (− 2.600, − 0.069), p = 0.063) (Table 2 and Fig. 2B). Here, the F-test result also showed no significant difference between the estimated β-coefficients of sham and active tVNS (F_1,72_ = 0.483, p = 0.506). BMI was neither in the young nor in the old cohort a significant covariate (young group: β_BMI_ = 0.280 (− 0.169, 0.730), p = 0.307; old group: β_BMI_ = − 0.099 (− 0.265, 0.067), p = 0.319).

**Table 2:** Shown are means ± standard deviations of the non-transformed [pg/ml] and Yeo-Johnson-transformed aldosterone concentrations for the younger and older cohort.

	Aldosterone (non-transformed)	Aldosterone (Yeo-Johnson)	β-coefficient (± 95%CI)	p-value
Young cohort				
Active Pre	132.25 ± 86.57	10.54 ± 2.59	− 1.610 (− 2.855, − 0.365)	0.022
Active Post	93.60 ± 70.40	8.93 ± 2.95		
Sham Pre	166.48 ± 89.74	11.61 ± 2.60	− 0.857 (− 2.102, 0.388)	0.257
Sham Post	139.28 ± 89.49	10.75 ± 2.63		
Old cohort				
Active Pre	125.16 ± 67.98	12.59 ± 3.05	− 1.969 (− 3.234, − 0.703)	0.005
Active Post	87.88 ± 51.60	10.62 ± 3.14		
Sham Pre	110.47 ± 65.26	11.77 ± 3.38	− 1.334 (− 2.600, − 0.069)	0.063
Sham Post	83.52 ± 48.62	10.44 ± 2.92		

Estimated β-coefficients are presented with the corresponding 95% confidence interval (95%CI) based on the Yeo-Johnson-transformed data.

### Young women had the highest aldosterone levels

**Figure 3:**
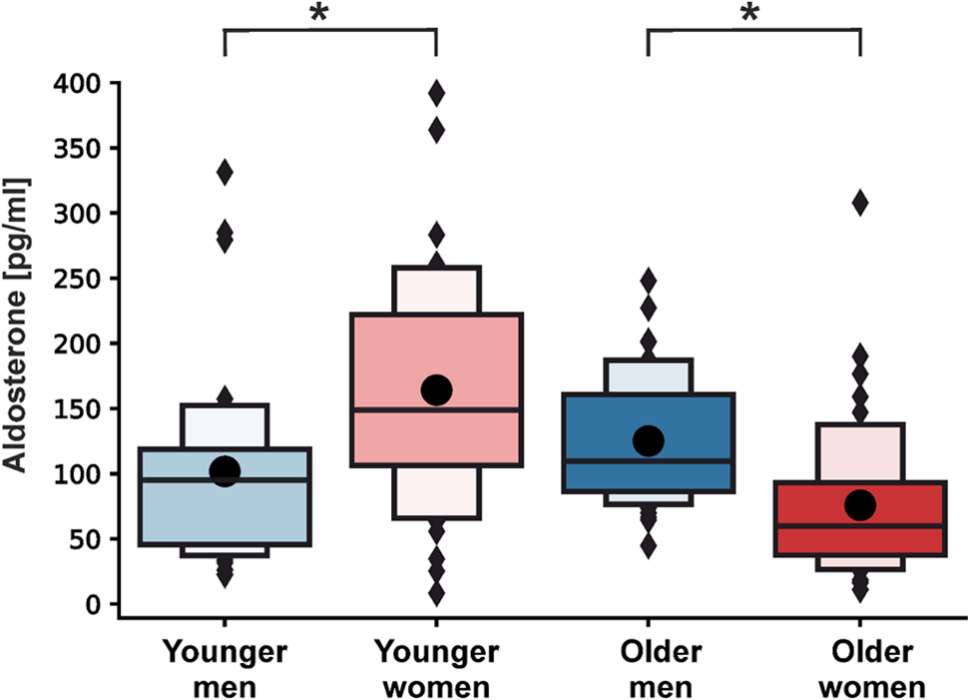
Distribution of serum aldosterone concentrations by age and sex. Shown are boxplots representing the distribution of serum aldosterone concentrations in pg/ml separately for males and females of the younger and older cohort. The darker-colored boxes pool 50% of all the data points, while together with the light-colored boxes, 75% of all the data points are depicted. The black lines depict the median of the respective distribution plots, while the black dots represent the respective mean. Significant differences are shown between male and females within a cohort based on the Yeo-Johnson transformed data set (linear mixed models). *p < 0.05.

Raw data of the aldosterone concentrations in the groups of younger and older men and women are shown in Table 3 and S1 Fig. Independent of active and sham tVNS, younger men showed significantly lower aldosterone concentrations compared to younger women (β_male_ = − 2.412 (− 4.258, − 0.566), p = 0.022, Fig. 3). Interestingly, opposite effects were observed in the older cohort, where males had significantly higher aldosterone levels compared to females (β_male_ = 3.600 (1.497, 5.702), p = 0.002) (Fig. 3). The LMM based on all aldosterone measurements revealed a significant interaction effect between sex and age (Table 4), demonstrating that young females of this sample had the highest aldosterone concentrations and old females the lowest.

**Table 3:** Raw data of aldosterone concentrations in male and female study participants during pre- and post-stimulation.

Aldosterone [pg/ml]	Young cohort		Old cohort	
	Women	Men	Women	Men
Active Pre	172.13 ± 101.95	92.38 ± 43.53	104.94 ± 85.80	143.37 ± 43.82
Active Post	101.35 ± 65.79	85.85 ± 77.46	68.50 ± 55.94	105.32 ± 42.79
Sham Pre	214.33 ± 82.27	118.62 ± 71.73	81.05 ± 53.76	136.95 ± 65.60
Sham Post	168.62 ± 90.84	109.94 ± 82.10	48.80 ± 27.53	114.77 ± 42.00

Shown are means ± standard deviations for the younger and older cohort.

**Table 4:** Results from a linear mixed model using the parameters body-mass index (BMI), sex, age and the interaction term of sex and age on Yeo-Johnson-transformed aldosterone concentrations irrespective of stimulation conditions (tVNS, sham).

	β-coefficient (± 95%CI)	p-value
Intercept (Young females)	11.488 (8.660, 14.317)	
Sex (Effect of being male)	− 1.755 (− 3.238, − 0.271)	0.038
Age (Effect of being old)	− 2.829 (− 4.383, − 1.276)	0.001
Interaction sex with age	4.108 (1.947, 6.269)	0.001
BMI	− 0.033 (− 0.158, 0.091)	0.599

Depicted are the estimated β-coefficients, including their corresponding 95% confidence interval (95%CI). The following dummy coding was applied: sex (female = 0, male = 1), age (younger = 0, older = 1).

## Discussion

This post hoc analysis demonstrated that, in univariate comparison, there was no significant difference between auricular tVNS at the tragus and sham treatment at the earlobe with respect to alterations in serum aldosterone concentrations from pre- to post-stimulation. In both younger and older participants, aldosterone levels decreased during the treatment period and, although this decline was more pronounced during active tVNS, the difference was not enough to conclude relevant effects compared to sham stimulation. Irrespective of the stimulation conditions, aldosterone concentrations depended significantly on the gender and age group. Among the study participants, younger women had the highest aldosterone levels, which were significantly elevated compared to younger men and older women, with the latter group having the lowest serum concentrations.

It is well known that the risk of developing cardiovascular diseases and hypertension differs between men and women^31–33^. While young, premenopausal women have a lower risk of hypertension compared to young men, the risk reverses after menopause^31,34^. The age-related aldosterone differences in women, but not in men, may be related to the decline in the levels of the two female sex hormones estrogen and progesterone after menopause. In this respect, studies in animals and humans have demonstrated that estrogen has modulatory effects on the RAAS by inhibiting its hypertensive axis while enhancing vasodilatory effects^34–36^.

For example, estrogen has been shown to reduce angiotensin-II-receptor 1 expression, while increasing the expression of angiotensin-converting enzyme 2, which stimulates angiotensin (1–7) production and angiotensin-II-receptor-2 activation^37^. Additionally, vasodilatory effects are also conveyed by nitric oxide production mediated via the estrogen receptor-α (ERα) and -β (ERβ) in endothelial cells^38^. Interestingly, in salt-sensitive but not salt-resistant rats, estrogen loss was associated with a reduction in renal ERα expression, suggesting a potential mechanism for postmenopausal hypertension in salt-sensitive individuals^39^. Moreover, ERβ signaling was shown to inhibit aldosterone secretion in human HAC15 adrenocortical cells in vitro, while its blockage resulted in aldosterone secretion through estrogen activation of G protein-coupled estradiol receptor-1 (GPER-1)^40^. Notably, aldosterone was found to interact with GPER-1, although the physiological effects of this non-classical pathway are still unknown^41^.

All these data suggest an inhibitory role of estrogen on RAAS in younger women, which would imply lower aldosterone concentrations compared to men. Consistent with this view, a population-based study with over 3000 participants reported lower plasma aldosterone levels in younger and older women compared with men^42^. Likewise, two studies with young, healthy subjects reported significantly lower aldosterone concentrations in premenopausal women compared to young men under a high-salt diet with maximally suppressed RAAS^43,44^. While serum aldosterone levels of women at baseline did not differ from those of men in 100 healthy, normotensive participants, the values in women were significantly reduced after a sodium loading test^45^. In contrast, data from our study showed the opposite effect, with younger women having significantly higher aldosterone levels than their male counterparts. There are also two more studies which found higher plasma aldosterone levels in female participants compared with male subjects^46,47^.

Furthermore, a drop in estrogen after menopause should have a stimulatory effect on aldosterone, which however was neither observed in the present nor in other studies. Consistent with our results, data from 2891 subjects enrolled in the Framingham Heart Community Study showed that postmenopausal women not using hormonal replacement therapy had significantly lower serum aldosterone levels than premenopausal women^46^. Similarly, a cross-sectional study in 442 patients with primary aldosteronism found a decrease in aldosterone levels in women older than 54 years compared to women younger than 46 years^47^. In a sample of 100 normotensive healthy subjects, women younger than 50 years had significantly higher baseline aldosterone levels than women older than 50 years^45^. Using liquid chromatography–tandem mass spectrometry in plasma samples from 525 subjects, Eisenhofer and colleagues demonstrated significantly higher aldosterone levels in premenopausal women with and without oral contraceptives compared to postmenopausal women^48^.

These discrepancies in results could be due to the influence of progesterone on aldosterone production during the female menstruation cycle. Interestingly, progesterone, but not estrogen, stimulated the secretion of aldosterone in zona glomerulosa cells from female rats in vitro^49^. Since progesterone is an antagonist for the mineralocorticoid receptor, higher levels of progesterone during the luteal phase may lead to a compensatory increase in renin and aldosterone^49,50^. Thus, it has been previously shown that women have increased serum aldosterone concentrations during a high-sodium diet in the luteal phase of their menstruation cycle^49–51^. Therefore, it is possible that at least some younger females in the present study were in the luteal phase, which resulted in an elevated aldosterone level compared with men and older women.

To the best of our knowledge, this is the first study to investigate tVNS effects on circulating aldosterone levels in humans. Two recent animal studies have reported that rats exposed to chronic mild stress showed significantly reduced circulating ACTH and corticosterone concentrations after auricular tVNS treatment compared to controls, indicating inhibition of the HPA axis and possibly aldosterone^52,53^. In addition, six weeks of bilateral tVNS at the tragus significantly blunted the increase of plasma aldosterone and norepinephrine, while also diminishing cardiac remodeling in 22 dogs with induced myocardial infarction^54^. However, the results of our post hoc analysis did not suggest an inhibitory effect of short-term tVNS on the serum aldosterone concentrations in healthy participants, since a drop in aldosterone levels was also present during sham stimulation of the earlobe.

Although the earlobe is intensively used as a sham location in tVNS studies, its usage is still under debate^55,56^. The earlobe is innervated by the superficial cervical plexus including the great auricular nerve, which has previously been shown in fMRI studies to activate structures of the limbic system similar to tVNS, albeit less intensely^57^. In addition, sham stimulation at the earlobe was able to elicit weak pupillary dilation, which is an indirect biomarker for norepinephrine release and possibly for vagal stimulation^58,59^. Therefore, it cannot be ruled out that earlobe stimulation influenced our results by producing effects similar to active tVNS. Another possible explanation for the decrease in aldosterone concentrations on both sham and active stimulation days is the influence of circadian rhythmicity. Aldosterone concentrations peak in the early morning with a steady decrease until midnight^60–63^. During this study, blood samples were collected for both experimental days at similar time points in the majority of subjects. Nevertheless, the period between two blood samples may have been long enough to represent circadian influences.

Several limitations must be considered when interpreting the results of this analysis. Firstly, the generalizability of our results is considerably limited, since the entire study cohort consisted of white participants and the sample size was very small, which particularly restricts the interpretability of ethnic and gender differences. Secondly, salt intake was not controlled, hence it cannot be excluded that significant differences in aldosterone levels were caused by prior sodium intake. Thirdly, detailed information about the menstrual cycle or menopausal status of our female participants was not available. Therefore, it cannot be excluded that increased progesterone concentrations in younger women may have caused their high aldosterone levels. Fourthly, the concentrations of potassium, angiotensin II, renin and ACTH were not assessed, restricting any conclusions on the effects of gender and tVNS treatment on the HPA axis and the RAAS. Furthermore, blood was drawn in a seated position, therefore aldosterone levels were probably higher than in a supine position^63,64^, limiting a direct comparison with other studies, in which aldosterone was measured in other positions. Additionally, due to the constrains associated with a post hoc analysis^65^, no causal conclusions can be drawn from this study. Finally, participants did not receive standardized stress tests, so it may be of interest to investigate whether tVNS modulates the RAAS under acute stress. Given these limitations, additional studies are required to verify our results.

## Conclusion

In summary, this post-hoc evaluation suggests that short-term auricular tVNS and sham treatment do not differ with respect to changes in serum aldosterone concentration in healthy subjects. Moreover, we observed an interaction effect of sex and age on circulating aldosterone levels in the entire study cohort, where younger women had the highest and older women the lowest concentrations. However, due to several limitations of this study, no direct conclusions can be drawn, and further studies on the effects of tVNS and sex differences in the regulation of RAAS components are required.

### Supplementary Information

Supplementary Information.

## Data Availability

The datasets used and analysed during the current study are available from the corresponding author on reasonable request.
