# Supplementary material for: A randomized vagus nerve stimulation study demonstrates that serum aldosterone levels decrease with age in women, but not in men
Source: Sci Rep. 2023 Aug 30;13:14197. doi: 10.1038/s41598-023-40113-9 (PMC10469189; doi:10.1038/s41598-023-40113-9)
Supplement: Supplementary file 1 — Supplementary Information. [file 41598_2023_40113_MOESM1_ESM.docx]

**S1 Table.** Estimated aldosterone concentrations in pg/ml for the younger cohort.

| **Subject** | **Gender** | **Before**  **active tVNS** | **After**  **active tVNS** | **Before**  **sham tVNS** | **After**  **sham tVNS** |
| --- | --- | --- | --- | --- | --- |
| 1 | female | 72.25503072 | 66.09333408 | 166.5855797 | 106.463083 |
| 2 | male | 149.0743692 | 72.82924364 | 123.6135249 | 95.66631032 |
| 3 | male | 45.91750234 | 26.02944516 | 152.6559218 | 112.3288455 |
| 4 | male | 157.351192 | 285.00267 | 146.0649805 | 331.3390354 |
| 5 | male | 105.4788541 | 92.16759976 | 279.4008434 | 103.6228276 |
| 6 | female | 141.6646849 | 124.1436737 | 158.4788285 | 106.482074 |
| 7 | female | 149.1233086 | 55.7981089 | 136.066006 | 146.7161051 |
| 8 | female | 95.34937392 | 120.2604305 | 111.6004252 | 221.3027395 |
| 9 | female | 207.1663465 | 148.5368799 | 228.2171695 | 363.7523569 |
| 10 | female | 232.159366 | 152.5312829 | 391.8248358 | 202.9343477 |
| 11 | female | 392.1818357 | 225.0103482 | 283.2819874 | 224.4556105 |
| 12 | female | 257.6693807 | 87.48789772 | 203.2907075 | 114.4633853 |
| 13 | female | 63.40878585 | 8.34232528 | 260.6364682 | 165.0130053 |
| 14 | female | 110.3092657 | 25.30146416 | 203.3204299 | 34.59044802 |
| 15 | male | 35.93563167 | 33.6376194 | 39.30574144 | 43.45134287 |
| 16 | male | 119.1671608 | 109.4058491 | 108.5621106 | 100.6124231 |
| 17 | male | 37.48594083 | 22.54203631 | 44.75845942 | 68.84604816 |
| 18 | male | 102.7365777 | 103.5158363 | 50.12763391 | 80.11699694 |
| 19 | male | 94.43484334 | 81.5784034 | 89.49276522 | 118.5649179 |
| 20 | male | 76.17376959 | 31.74504878 | 152.24978 | 44.82576485 |

**S2 Table.** Yeo-Johnston transformed aldosterone concentrations for the younger cohort.

| **Subject** | **Gender** | **Before**  **active tVNS** | **After**  **active tVNS** | **Before**  **sham tVNS** | **After**  **sham tVNS** | **BMI** |
| --- | --- | --- | --- | --- | --- | --- |
| 1 | female | 8.29769708 | 8.009786605 | 11.3859569 | 9.63935854 | 20.8 |
| 2 | male | 10.9317156 | 8.323626022 | 10.1974732 | 9.25417646 | 22.9 |
| 3 | male | 6.9079485 | 5.410884665 | 11.0276153 | 9.83703156 | 19.2 |
| 4 | male | 11.1509253 | 13.79534584 | 10.8498525 | 14.5394584 | 24.4 |
| 5 | male | 9.60543334 | 9.12271893 | 13.6996238 | 9.54083832 | 25.9 |
| 6 | female | 10.7279514 | 10.21381779 | 11.1801467 | 9.64001093 | 19.4 |
| 7 | female | 10.933037 | 7.482943667 | 10.5688698 | 10.8676664 | 18.6 |
| 8 | female | 9.24241079 | 10.09291885 | 9.81289126 | 12.6147344 | 21.0 |
| 9 | female | 12.3202856 | 10.91718183 | 12.7538601 | 15.0165553 | 22.8 |
| 10 | female | 12.8318318 | 11.02430513 | 15.4056428 | 12.2293405 | 23.3 |
| 11 | female | 15.4104598 | 12.68971706 | 13.7660883 | 12.6785547 | 19.6 |
| 12 | female | 13.3146861 | 8.941205365 | 12.2370508 | 9.90713356 | 23.2 |
| 13 | female | 7.87836363 | 3.102550469 | 13.3685849 | 11.3466032 | 21.8 |
| 14 | female | 9.76982339 | 5.342588449 | 12.2376935 | 6.12903513 | 20.8 |
| 15 | male | 6.23015638 | 6.055729095 | 6.47233098 | 6.75097608 | 21.0 |
| 16 | male | 10.0583789 | 9.739474487 | 9.7109683 | 9.43428866 | 22.7 |
| 17 | male | 6.34345709 | 5.070617268 | 6.83493484 | 8.14066807 | 26.0 |
| 18 | male | 9.50970263 | 9.537089622 | 7.16270385 | 8.64073133 | 19.8 |
| 19 | male | 9.20830346 | 8.701824924 | 9.01979614 | 10.0392553 | 22.9 |
| 20 | male | 8.47182961 | 5.90569924 | 11.0168219 | 6.83921096 | 21.9 |

**S3 Table.** Estimated aldosterone concentrations in pg/ml for the older cohort.

| **Subject** | **Gender** | **Before active tVNS** | **After active tVNS** | **Before sham tVNS** | **After**  **sham tVNS** |
| --- | --- | --- | --- | --- | --- |
| 1 | female | 58.2243727 | 11.0714203 | 57.6655749 | 18.3658071 |
| 2 | female | 41.3681607 | 39.0980036 | 91.4723843 | 39.5827427 |
| 3 | male | 171.931747 | 44.8136061 | 77.1427091 | 76.984836 |
| 4 | female | 118.728005 | 190.043101 | 26.3996093 | 29.4161637 |
| 5 | female | 66.9956371 | 65.5826861 | 16.4622512 | 28.6186759 |
| 6 | female | 159.033778 | 61.5333915 | 176.497417 | 81.4052484 |
| 7 | male | 186.976884 | 159.097746 | 81.5103065 | 91.2314636 |
| 8 | male | 162.194931 | 156.139853 | 247.998459 | 201.561408 |
| 9 | male | 187.697891 | 66.4640585 | 180.388878 | 87.5083542 |
| 10 | female | 98.8768516 | 69.597221 | 84.2973787 | 66.2466561 |
| 11 | male | 114.089938 | 123.709736 | 64.5054871 | 96.1076425 |
| 12 | female | 40.297689 | 25.3161565 | 39.3321509 | 26.59261 |
| 13 | male | 129.636276 | 88.1489992 | 144.520395 | 103.832359 |
| 14 | male | 201.048793 | 159.284089 | 227.201628 | 177.20883 |
| 15 | female | 52.9711334 | 32.2808802 | 90.2449858 | 51.102481 |
| 16 | male | 92.7708712 | 73.7795342 | 91.4769077 | 103.633564 |
| 17 | female | 307.92677 | 121.982204 | 147.073517 | 97.8359364 |
| 18 | male | 107.3548 | 111.87785 | 160.221906 | 126.350095 |
| 19 | male | 79.9956768 | 69.9112652 | 94.5518302 | 83.2492149 |

**S4 Table.** Yeo-Johnston transformed aldosterone concentrations for the older cohort.

| **Subject** | **Gender** | **Before active tVNS** | **After active tVNS** | **Before sham tVNS** | **After**  **sham tVNS** | **BMI** |
| --- | --- | --- | --- | --- | --- | --- |
| 1 | female | 7.61294311 | 3.59841851 | 7.5833478 | 4.61145487 | 24.4 |
| 2 | female | 6.61341285 | 6.45783426 | 9.09617529 | 6.49157762 | 31.2 |
| 3 | male | 11.5177864 | 6.83843882 | 8.5138988 | 8.50707004 | 25.8 |
| 4 | female | 10.0444408 | 11.9436481 | 5.44510455 | 5.71224595 | 21.5 |
| 5 | female | 8.05310733 | 7.9850834 | 4.37813007 | 5.64354921 | 18.8 |
| 6 | female | 11.1944729 | 7.78419169 | 11.6280637 | 8.69462714 | 23.6 |
| 7 | male | 11.8736598 | 11.1961219 | 8.69899554 | 9.08694328 | 28.7 |
| 8 | male | 11.2754033 | 11.1193674 | 13.1358552 | 12.1995442 | 27.4 |
| 9 | male | 11.8901908 | 8.02763607 | 11.7204538 | 8.94201371 | 25.0 |
| 10 | female | 9.37182252 | 8.17573638 | 8.81344776 | 8.01717728 | 27.9 |
| 11 | male | 9.8949367 | 10.2004432 | 7.9325213 | 9.27051385 | 49.1 |
| 12 | female | 6.54081981 | 5.34398021 | 6.47417007 | 5.46281452 | 29.0 |
| 13 | male | 10.3802877 | 8.96726108 | 10.8073662 | 9.54817178 | 28.1 |
| 14 | male | 12.1883818 | 11.200923 | 12.7336175 | 11.6450622 | 24.6 |
| 15 | female | 7.32632332 | 5.94879709 | 9.04895941 | 7.21952056 | 21.3 |
| 16 | male | 9.14563666 | 8.36621949 | 9.09634846 | 9.54121435 | 29.4 |
| 17 | female | 14.1735436 | 10.146863 | 10.8774207 | 9.33398204 | 18.6 |
| 18 | male | 9.66990214 | 9.82209852 | 11.2250248 | 10.2813109 | 30.0 |
| 19 | male | 8.63562403 | 8.19031776 | 9.21267958 | 8.77072524 | 25.7 |


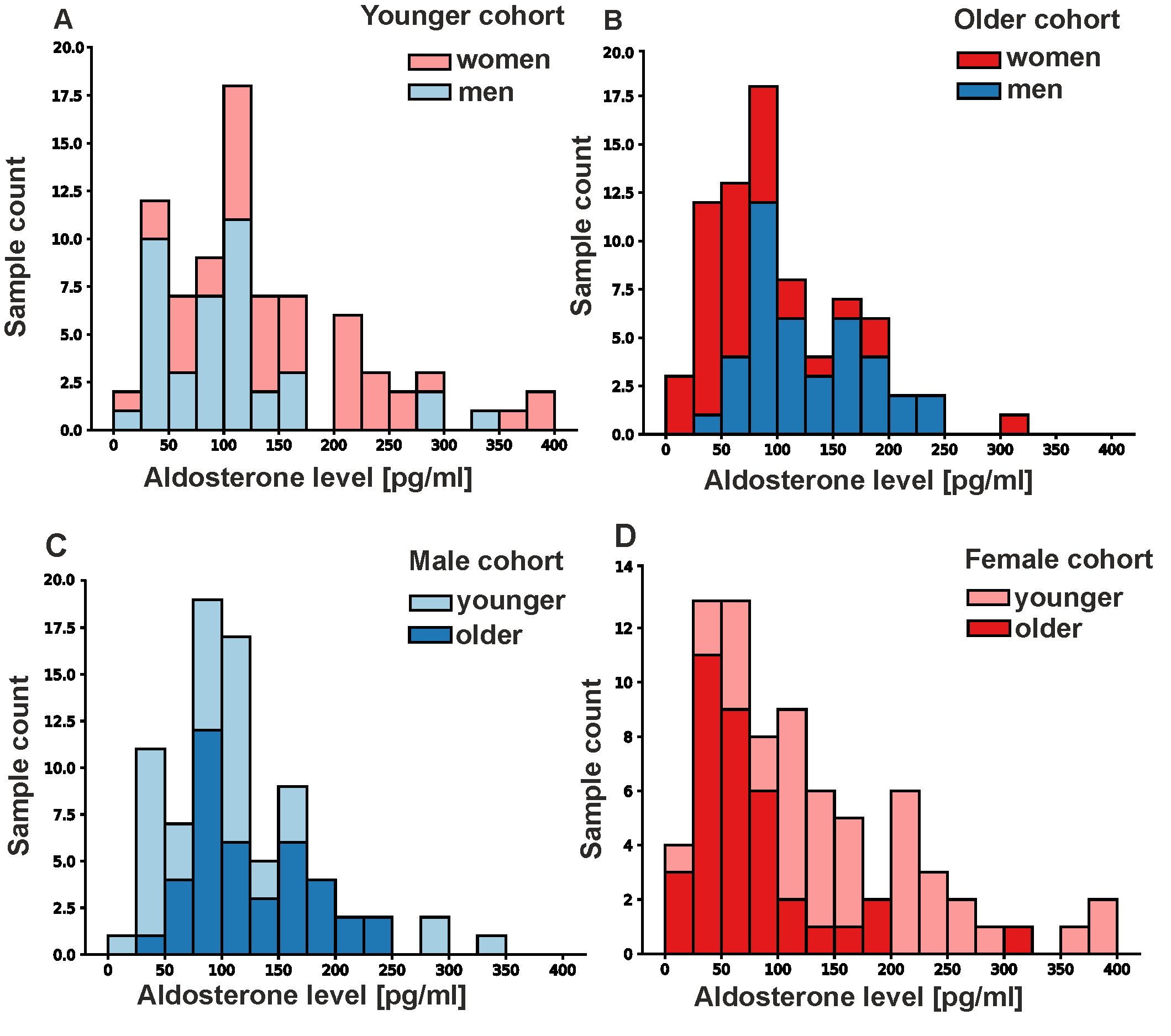


**Supplemental Figure 1. Histograms of serum aldosterone concentrations by age group and sex.** Distribution of aldosterone levels in the young cohort (n=10 males, n=10 females) (**A**), in the older males (n=10) and females (n=9, **B**), and separately in all male (**C**) and female (**D**) study participants.
